# Innate Immune System Biomarkers in Asthma in Older Age: A Mini Review

**DOI:** 10.34172/aim.34410

**Published:** 2026-02-01

**Authors:** Fatemeh Zamani, Rasoul Nasiri Kalmarzi, Khaled Fathizadeh, Naseh Sigari, Sabah Hasani, Ramin Lotfi, Abbas Khosravi

**Affiliations:** ^1^Lung Diseases and Allergy Research Center, Research Institute for Health Development, Kurdistan University of Medical Sciences, Sanandaj, Iran; ^2^Department of Pediatrics, Faculty of Medicine, Kurdistan University of Medical Sciences, Sanandaj, Iran; ^3^Department of Internal Medicine, Faculty of Medicine, Kurdistan University of Medical Sciences, Sanandaj, Iran; ^4^Blood Transfusion Research Center, High Institute for Research and Education in Transfusion Medicine, Tehran, Iran; ^5^Kurdistan Regional Blood Transfusion Center, Sanandaj, Iran; ^6^Clinical Research Development Center, Tohid Hospital, Kurdistan University of Medical Sciences, Sanandaj, Iran; ^7^Department of Education, Faculty of teachers Education Farhangian University, Gorgan, Iran

**Keywords:** Aging, Asthma, Eosinophil, Innate immunity

## Abstract

Asthma in older adults represents a growing and underrecognized clinical challenge, characterized by increased morbidity, mortality, and complex immunological alterations associated with aging. Although the global literature on asthma immunopathogenesis continues to expand rapidly, particularly in the areas of immunosenescence, inflammaging, and biomarker discovery, a focused synthesis of innate immune mechanisms specific to older populations remains limited. Epidemiological data indicate that asthma prevalence in individuals aged≥60 years ranges from 4% to 13%, yet this group accounts for a disproportionate share of asthma-related mortality. Age-associated physiological changes, comorbidities, and frequent underdiagnosis contribute to poorer outcomes. Emerging evidence from academic research centers highlights profound age-related alterations in epithelial integrity, neutrophil and macrophage function, cytokine regulation, and innate immune receptor signaling, resulting in asthma phenotypes that differ fundamentally from those seen in younger patients. In parallel, advances in biomarker research (including inflammatory mediators, cellular signatures, and epigenetic markers) offer new opportunities for improved phenotyping and precision management in elderly asthma. This mini-review synthesizes contemporary findings from reputable academic studies to summarize key innate immune alterations and candidate biomarkers relevant to asthma in older adults. By consolidating current evidence within a rapidly evolving field, this review aims to provide a clinically meaningful framework for researchers and clinicians seeking to better understand immune aging in asthma and to guide future investigation and targeted therapeutic strategies.

## Introduction

 Asthma in older adults is increasingly recognized as a distinct clinical and immunological entity rather than a simple extension of the disease observed in younger populations. While the volume of published research on asthma pathogenesis and immune regulation continues to grow at an unprecedented pace, particularly in the fields of aging biology and systems immunology, the integration of these findings into a coherent framework specific to elderly asthma remains incomplete. Academic studies from leading research institutions have consistently demonstrated that aging profoundly reshapes innate immune responses, airway structure, and inflammatory regulation, resulting in phenotypes that are frequently non-atopic, neutrophilic, and less responsive to corticosteroids.^1^ In this context, periodic synthesis of evolving evidence is essential. Summarizing and contextualizing emerging data allow for identification of consistent patterns, clinically relevant biomarkers, and unresolved knowledge gaps. This review, therefore, focuses on consolidating current high-quality evidence on innate immune system alterations in asthma among older adults, with particular emphasis on biomarkers that may inform diagnosis, phenotyping, and therapeutic decision-making in this expanding patient population.^2^ Age-related changes in lung function, alongside infectious comorbidities, increase the overall burden of asthma on the health and quality of life for older adults. This necessitates a holistic approach integrating geriatric principles in managing asthma.^3^ The presentation, diagnosis, and management of asthma become more complicated with age.^4,5^ Structural changes in the aging lung, compounded by asthma-related alterations, can worsen disease severity and impair lung function.^6^ Aging also affects cellular composition and immune function in the airways, processes that are less understood than their counterparts in younger individuals.^7^ Aging is accompanied by a multifaceted remodeling of the immune system, broadly encompassed under the term immunosenescence. This encompasses a decline in the efficacy of both innate and adaptive immune responses. This immunological milieu, marked by senescence, is characterized by decline in naïve T cell production, contraction of the T cell receptor repertoire, and accumulation of memory and senescent T cells.^8^ This process impairs antigen-specific adaptive immunity. Concurrently, the innate immune system emerges as a pivotal protagonist, manifesting maladaptations such as diminished mucociliary clearance, altered function of airway neutrophils and macrophages, and skewed cytokine production with a pronounced chronic low-grade inflammatory state termed “inflamm-aging.” This chronic inflammatory process is characterized by elevated systemic and airway levels of pro-inflammatory cytokines, including interleukin IL-6, tumor necrosis factor-alpha (TNF-α), and IL-8, which contribute to persistent airway inflammation distinct from classical allergic asthma. The specific phenotypes of asthma in the aging population are not clearly defined. However, the age of onset and the overlap with chronic obstructive pulmonary disease (COPD) may influence disease characteristics.^4^ Diagnostic tests for asthma in older adults generally utilize the same methods as younger patients, though modifications may be necessary to account for comorbidities, cognitive impairments, and the adverse effects of medications.^9^ Future research should prioritize developing age-specific methods for diagnosing, monitoring, and treating asthma in older populations. This review explores innate immune system biomarkers in asthma, focusing on the importance of shared decision-making in optimizing care for older adults. In addition to immune and structural changes in the lung with aging, epigenetics (including DNA-methylation changes, histone modifications, and noncoding RNAs) plays an important role in regulating the innate immune response and inflammatory patterns in asthma in the elderly. Recent evidence suggests a correlation between methylation profiles and epigenetic markers of aging with asthma severity and inflammatory markers. This correlation may support the proposal of these biomarkers as a means of diagnosis and as therapeutic targets.

## Summary of Key Points

- The relationship between phenotypes, genotypes, and endotypes in the elderly population: The interplay among phenotypes, genotypes, and endotypes in elderly individuals illuminates the manner in which observable symptoms, genetic composition, and underlying biological mechanisms converge to give rise to disease patterns associated with the aging process.
- Chronic Low-Grade Inflammation: Characterized by “inflamm-aging,” leading to increased pro-inflammatory cytokines (e.g. TNF-α, IL-6).
- Impaired Epithelial Barrier Function: Decline in integrity and regenerative capacity of airway epithelium, increasing vulnerability to allergens and pathogens.
- Senescence-Associated Secretory Phenotype (SASP): Senescent cells secrete pro-inflammatory cytokines and chemokines, contributing to chronic inflammation.
- Altered Complement System: Changes in complement factors affect inflammatory processes and immune responses.
- Changes in Pattern Recognition Receptors (PRRs): Alterations in TLRs and NLRs affect allergen recognition and immune responses, increasing susceptibility to exacerbations.
- Dysregulated Cytokine Production: Variations in cytokines like IL-6 and IL-8 impact airway inflammation and asthma control.

## Asthma Phenotypes, Genotypes, and Endotypes in the Elderly Population

 Asthma is now widely recognized as a heterogeneous disorder, characterized by variation in clinical expression (phenotypes), genetic susceptibilities (genotypes), and underlying mechanistic pathways (endotypes).^10^ Across the lifespan, these dimensions diverge substantially: childhood- and early-onset asthma is typically atopy-driven, associated with eosinophilic T2-high inflammation and strong genetic predisposition, whereas late-onset and elderly asthma frequently manifests non-atopic, neutrophilic T2-low pathways with diminished IgE and reduced corticosteroid responsiveness.^11^

 The process of aging profoundly influences asthma biology through immunosenescence and inflammaging, which alter epithelial integrity, impair mucociliary clearance, and disrupt cytokine homeostasis, thereby enhancing susceptibility to infection and chronic airway inflammation.^9,12,13^ Neutrophilic airway inflammation is more prevalent in elderly asthmatics. Increased sputum neutrophil counts and elevated levels of neutrophil-derived biomarkers, such as IL-8, have been found to correlate with greater disease severity and steroid resistance in this group.^14^ Furthermore, there is evidence of dysregulated activation of innate immune receptors, such as toll-like receptors (TLRs), and enhanced interferon pathway signaling, particularly in neutrophilic asthma. This suggests the potential for persistent innate immune activation, possibly driven by the presence of subclinical infections or alterations to the microbiome.^15^ Furthermore, the process of aging has been linked to immunosenescence, a term denoting the functional impairment of innate immune cells, including macrophages, dendritic cells, and natural killer cells. This impairment is characterized by a reduction in phagocytic activity, alterations in cytokine profiles, and a decrease in antigen presentation capacity.^16^ These changes contribute to persistent neutrophilic airway inflammation, elevated neutrophil-derived biomarkers such as IL-8, and steroid resistance in elderly asthma.^17^ Dysregulated innate immunity (including aberrant toll-like receptor signaling and heightened interferon pathway activity) likely sustains this phenotype, with evidence implicating subclinical infection and microbiome alterations as additional drivers.^18^

 Clinically, elderly asthma patients display a lower prevalence of atopy, reduced eosinophilia, diminished IgE, more pronounced fixed airflow limitation, and a higher prevalence of comorbid chronic obstructive pulmonary disease (COPD).^19^ Latent class and cluster analyses confirm multiple phenotypic subsets in this population, including neutrophilic versus eosinophilic inflammatory profiles, as well as obesity- or smoking-related clusters.^20^ Importantly, biomarker studies indicate that severe elderly asthma is associated with elevated eotaxin-2 and transforming growth factor-β1 (TGF-β1), which contribute to airway remodeling and fibrosis, underscoring their relevance as both markers of disease activity and potential therapeutic targets.^6^

 Recent advancements in multi-omics and biomarker discovery are beginning to clarify these complex relationships by delineating molecular signatures that distinguish endotypes, enabling more precise stratification and paving the way toward personalized, phenotype–genotype–endotype-based therapeutic strategies.^21,22^ Such integration of genotypic and phenotypic data is consistent with global precision medicine initiatives and remains essential for advancing effective and individualized asthma management in the elderly.

## Chronic Low-grade Inflammation

 The innate immune system is vital for maintaining pulmonary homeostasis, but aging leads to immune changes that influence inflammation. “Inflamm-aging” describes the increased levels of pro-inflammatory cytokines like TNF-α and IL-6, observed even without systemic inflammatory disorders or infections.^2^ Such changes manifest in the lungs with increased neutrophils and elevated IL-6 in the bronchoalveolar lavage fluid (BALF), often in the absence of apparent airway disease. This chronic low-grade inflammation is closely associated with immune senescence, evidenced by the accumulation of senescent cells and irregularities in cytokine production. Such immune-related changes can facilitate airway inflammation and contribute to asthma pathophysiology. Reliable biomarkers like C-reactive protein (CRP), IL-6, and TNF-α serve as indicators of inflammation severity in older adults with asthma.^6,23^ Notably, disparities in inflammatory profiles between younger and older individuals with asthma relate directly to elevated sputum neutrophils, eosinophils, and cytokines linked to neutrophil recruitment. These differences in airway inflammation can significantly impact asthma management for aging.^2,24^ Certain inflammatory markers have clinical implications; elevated levels of sputum IL-6 and MIP-3α/CCL20 in older patients correlate with reduced asthma control. In contrast, increased sputum neutrophils, IL-1β, IL-6, and MIP-3α/CCL20 are associated with elevated hospitalization risk. This suggests that specific age-related inflammatory markers provide critical insights into the clinical features of asthma.^25^ Cytokines such as IL-6 and IL-8 also interact intricately with TH17 cells, which may contribute to age-related disparities in sputum neutrophil values and clinical control of asthma. For example, IL-6 influences the differentiation of naive T cells into TH17 rather than Treg cells.^26^ Through multiple mechanisms, TH17 cells facilitate neutrophilia, particularly in younger asthmatics who exhibit reduced responsiveness to inhaled corticosteroids.^2^

## Senescence-associated Secretory Phenotype (SASP)

 Accumulating senescent cells exhibit a senescence-associated secretory phenotype (SASP), involving pro-inflammatory cytokine and chemokine secretion. SASP components in the airways of aging individuals with asthma may contribute to chronic inflammation and airway remodeling.^3,6^ The literature highlights the impact of cellular senescence, SASP, and their connection to asthma in older adults.

## Complement System

 The complement system, integral to the innate immune response, involves various components implicated in inflammatory processes, metabolism, and apoptosis. In the lungs, these components are synthesized locally, allowing for quick activation and inflammation onset.^27^ Aging correlates with a decline in immune efficacy, increasing susceptibility to respiratory infections.^28^

 Complement factors are potential therapeutic targets in asthma, particularly given the Th2-skewed inflammation associated with elevated pulmonary IL-4, IL-5, and IL-13 levels.^27^

## Fractional Exhaled Nitric Oxide (FeNO)

 Fractional exhaled nitric oxide (FeNO) is a non-invasive biomarker reflecting airway inflammation. Changes in FeNO levels warrant careful interpretation in older people with asthma. Elevated FeNO levels correlate with exacerbation risk and treatment response, suggesting significant implications for disease management in aging populations.^29^ Research indicates that while FeNO levels in stable older asthmatics are not consistently elevated, their interpretation may differ based on individual health factors, including atopy.^30^ Routine FeNO measurements may thus require additional context for clinical usefulness in geriatric asthma management.

## Impaired Epithelial Barrier Function

 Cellular senescence in airway epithelial cells contributes to impaired respiratory function in asthma.^31^ Aging results in reduced epithelial integrity and regenerative capability, which can be worsened by chronic inflammation and oxidative stress.^6,32^ This compromised barrier function heightens susceptibility to allergens and pathogens, increasing asthma exacerbations and respiratory infection risks in older adults. Research indicates that genes regulating epithelial barrier function, such as CDH1, are downregulated with age, reflecting significant implications for respiratory health.^33^

 Additionally, diminished levels of E-cadherin, another crucial protein involved in barrier function, have been observed in the airway epithelium of individuals with chronic obstructive pulmonary disease (COPD), likely contributing to documented epithelial barrier dysfunction in COPD and other airway diseases such as exacerbated asthma and respiratory infections.^34,35^

 Studies conducted on mice demonstrate that the ciliary beat frequency within the airway epithelium, vital for maintaining proper airway clearance, diminishes with age. This finding highlights age-related changes in the respiratory system. The loss of physical barrier function also increases susceptibility to bacterial and viral infections by facilitating their entry into deeper layers of the airway.^36^

 The loss of physical barrier function can contribute to heightened susceptibility to bacterial and viral infections, facilitating their access to submucosal layers. This increases an individual’s vulnerability to asthma.^37^ It is essential to highlight that respiratory tract infections in aging individuals significantly trigger asthma; for example, 90% of fatal cases of *C. difficile* colitis occur in individuals aged 65 years and older. Treatments for nasal polyps, including antibiotics, steroids, and surgery, are often inadequate, resulting in diagnoses of recalcitrant chronic rhinosinusitis (CRS) and nasal polyps. Such CRS and nasal polyps are associated with severe and uncontrolled asthma, representing another concern for the aging population.^37^

## Factors of the Innate Immune System

###  Acute Phase of Innate Immunity

 Aging alters the lung’s innate immune response, specifically through pattern recognition receptors (PRRs), which recognize pathogen-associated and damage-associated molecular patterns. Age-related changes in PRRs impact asthma, promoting airway inflammation and remodeling.^38,39^

 Research shows that dysregulated expression and function of PRRs, particularly Toll-like receptors (TLRs) and Nod-like receptors (NLRs), impair immune responses, increasing exacerbation risks in older asthmatics.^40^ Studies indicate that the response of TLR4, a key Toll-like receptor, to LPS is diminished in the peripheral blood monocytes of older individuals, leading to reduced interleukin-6 (IL-6) production. In older individuals with asthma, decreased expression of NLRP3, a member of the NLR family, in bronchial epithelial cells may hinder inflammasome activation, resulting in defective cytokine secretion and abnormal immune responses.^41^ Furthermore, human rhinovirus (hRV) infection in chronically inflamed nasal mucosa is associated with epithelial mucus hyperproduction via the major airway epithelial mucin (MUC5AC) production axis. The NLRP3 inflammasome pathway suppresses hRV replication in the airway epithelium.^42^

 RIG-I-like receptors, involved in recognizing viral RNA and activating antiviral responses, also exhibit age-related changes linked to altered immune responses against viral infections in asthma. Specifically, a decrease in RIG-I expression in airway epithelial cells of older individuals with asthma indicates impaired viral recognition and compromised antiviral defense mechanisms. This deficiency may increase susceptibility to viral-induced asthma exacerbations in older individuals.^43,44^

 C-type lectin receptors (CLRs) are crucial for the innate immune response against respiratory pathogens. Reduced expression of Dectin-1, a specific CLR, in the dendritic cells of older individuals with asthma may impair fungal pathogen recognition and clearance, leading to dysregulated immune responses and increased vulnerability to fungal-induced asthma exacerbations in elderly individuals.^45^ There is significant cross-regulation between CLRs and TLRs. The C-type lectin DC-SIGN modulates TLR signaling via Raf-1 kinase-dependent acetylation of the transcription factor nuclear factor-κB (NF-κB), influencing the polarization activity of naive T cells towards a Th2 cytokine response.^36,46^

## Innate Immune Cells

 The field of personalized medicine in asthma care has benefited from recognizing that “asthma” encompasses different clinical presentations. The goal is to link clinical phenotype to molecular mechanisms, defining an “endotype” that predicts therapy response.^47^ Earlier phenotyping methods focused on assessing sputum cellularity as an indirect measure of airway inflammation, identifying four subgroups of adult asthmatics: eosinophilic asthma, neutrophilic asthma, mixed granulocytic asthma, and paucigranulocytic asthma. Numerous studies report that eosinophilic (40-50%) and paucigranulocytic (30-50%) airway inflammation is prevalent among asthmatics, with neutrophilic asthma occurring in a smaller percentage (10-20%).^48,49^

## Neutrophils

 In response to priming agents such as endotoxin, granulocyte-macrophage colony-stimulating factor (GM-CSF), platelet-activating factor (PAF), and pro-inflammatory cytokines including tumor necrosis factor-alpha (TNFα), the neutrophils’ lifespan increases, thereby enhancing their bactericidal capacity.^50^

 The literature consistently indicates that the total number of circulating neutrophils does not change with age. Additionally, bone marrow cells from both young and aged subjects respond similarly to stem cell stimulants such as GM-CSF and interleukin IL-3. However, chemotaxis of neutrophils primed with GM-CSF towards the formylated tripeptide N-formyl-methionyl-leucyl-phenylalanine (fMLP) is reduced in cells from healthy aged volunteers compared to younger counterparts.^51^ Data suggest that changes in membrane fluidity associated with aging could provide a mechanism for many dysfunctional signaling pathways in neutrophils from aged individuals.^52,53^

 Generally, the aging process is linked with heightened lung inflammation even in the absence of lung disease. Bronchoalveolar lavage fluid (BALF) from individuals aged 19 to 83 years without allergies, pulmonary disease, or gastroesophageal reflux shows an increase in BALF neutrophils and CD4 + T cells with age, differing from alterations observed in peripheral blood (e.g. CD4 + T-cell counts may decrease with age). Individuals over 55 years with asthma, who exhibit spirometry reversibility and have less than 5 pack-years of smoking history, demonstrate higher sputum neutrophil counts compared to younger individuals with asthma. Among 14 older people with asthma studied, 13 were atopic, as defined by at least one positive skin prick test response to an aeroallergen.^54^

 Elevated airway neutrophilia correlates with heightened levels of sputum neutrophil mediators such as matrix metalloproteinase, neutrophil elastase, and IL-8 in older individuals with asthma, similar to changes observed in severe asthma characterized by neutrophilic dominance. This increase in airspace neutrophil counts may exacerbate asthma severity in aging populations.^55^

## Macrophage

 Research indicates that aging alters the biological properties of macrophages, potentially impacting asthma pathogenesis in older individuals. Aged macrophages may exhibit decreased chemotaxis and phagocytosis, essential functions in the immune response.^56^ These defects in aged macrophages have been linked to the effects of sex hormones and reproductive aging. Additionally, age-related changes in both the number and function of resident macrophages may contribute to increased susceptibility to asthma among the elderly.^16,57^ Investigations into intracellular signaling pathways reveal impairments in aged macrophages compared to their younger counterparts. Notably, reductions in MAP kinases such as ERK, p38, and JNK have been observed both before and after stimulation, potentially affecting immune responsiveness. Additionally, alterations in the expression and activation of NF-κB pathway members indicate a dysregulation of critical signaling pathways, suggesting that age-related modifications may favor asthma development. Contradictory data exist regarding the effects of aging on macrophage function. For instance, aged rodents exhibit greater lung and systemic effects following inhalation of airborne particulate matter compared to younger ones. A study reported reduced COX-2 protein levels in alveolar macrophages from aged rats after exposure to ozone reaction products, which may explain their increased susceptibility to air pollutants. Elevated COX-2 levels are associated with increased production of prostaglandin E2 (PGE2), which can inhibit MHC class II expression and IL-12 synthesis in CD8 + T cells.^58^

 Further studies have shown that aged mice display reduced levels of MAP kinases ERK, p38, and JNK in macrophages before and after lipopolysaccharide (LPS) stimulation. Additionally, differences in isoform expression between juvenile and senior rats suggest that aging affects MAP kinase functionality. For example, alveolar macrophages from young rats increase ERK levels following exposure to limonene ozone reaction products, while aged macrophages show a decrease.^58^

 Macrophages are crucial for innate immune responses within the alveolar compartment. They can either induce inflammation (M1 phenotype) or facilitate wound healing (M2 phenotype), depending on environmental signals. However, during inflammaging (a process characterized by chronic low-grade inflammation) macrophages lose their plasticity, impairing their ability to switch between pro- and anti-inflammatory states. Age-related changes include reduced production of pro-inflammatory cytokines (e.g. IL-6, IL-1β, TNFα), impaired phagocytosis, and decreased expression of Toll-like receptors (TLRs). These transformations may sustain inflammaging within the aging lung.^57,59^

## Natural Killer Cells and NKT Cells

 Natural killer (14) cells and natural killer T (NKT) cells are integral components of the immune system, particularly in antiviral defense. Research involving 82 subjects aged 30 to 99 years indicates that while NK cell numbers (CD3- CD161 + CD56 + ) increase with age, their cytotoxicity, as measured by ^51Cr release, declines on a per-cell basis. Conversely, NKT cell numbers (CD3 + CD161 + CD56 + ) decrease in older individuals, and their proliferation in response to interleukin-2 (IL-2) and α-GalCer is also diminished. The implications of these changes for asthmatic patients remain unclear, though there is growing interest in the roles of NK and NKT cells in asthma pathogenesis.^60^

 Previous studies have demonstrated a stark decline in NK cell activity with aging. For instance, NK cell activity is nearly absent in rodents by 25 months of age compared to 8 weeks. Interestingly, centenarians exhibit preserved NK cell cytotoxicity alongside increased NK cell numbers, although age-related changes have been linked to reduced proliferation rates and heightened susceptibility to infections and atherosclerosis. Despite the increase in NK cell numbers, there is a modest decline in their cytotoxic capabilities against NK-sensitive targets; however, their antibody-dependent cell-mediated cytotoxicity (ADCC) remains intact. Additionally, aged NK cells show impaired production of interferon-gamma (IFN-γ) and reduced responsiveness to IL-2.^61^

 NKT cells are characterized by their invariant T-cell receptor (TCR) that recognizes lipid antigens. They play critical roles in antiviral responses, anti-tumor immunity, and immune regulation.^62^ Although NKT cells influence both antigen-presenting cells and T-cell functions, their contribution to immunosenescence has been minimally studied. The role of invariant NKT (iNKT) cells in asthma remains controversial; some studies associate iNKT cells with asthma symptoms while others find no significant differences in airway eosinophilia between iNKT-deficient and wild-type mice. Generally, it is accepted that the absolute number of NKT cells increases with age, though the mechanisms behind this rise, whether due to longer lifespan or active expansion, remain unclear.^63^

 Systemic inhibition of NKT cell activation has been shown to prevent age-related declines in T-cell responses. NKT cells contribute to increased levels of immunosuppressive cytokines such as IL-10 and IL-4 with age while showing a decrease in IFN-γ production.^64^ These alterations suggest that aging negatively impacts NKT cell-mediated cytotoxic activity.

 There is increasing interest in the role of NKT cells within the context of asthma pathophysiology, particularly regarding immune senescence and inflammaging. Some studies propose that NKT cells may either exacerbate or alleviate asthma symptoms through cytokine production. For example, the removal of iNKT cells has been shown to prevent airway hyperactivity and pulmonary eosinophilic inflammation in murine models. Conversely, iNKT cells producing IL-4 and IL-13 are critical for allergen-induced airway inflammation. In summary, while NK and NKT cells are pivotal in shaping immune responses, aging appears to alter their functionality significantly. Further investigations are warranted to elucidate their roles in asthma development and exacerbation among older individuals.^63^

## Mast Cell

 Mast cells constitute a critical component in the pathophysiology of allergic asthma, primarily by contributing to airway inflammation and bronchoconstriction through the release of various pro-inflammatory mediators. Age-related alterations in immune function, particularly those affecting mast cell numbers and functionality, may significantly influence the severity and persistence of asthma in older adults. Notably, studies have indicated that changes in mast cell density and activity are tissue-specific and can impact the development and severity of asthma among aging populations.^65^

 In various asthma phenotypes, mast cells play an integral role in the initial phase of bronchoconstriction and the subsequent influx of inflammatory cells. Research findings illustrate that the effect of aging on mast cell parameters varies by tissue type and species. For instance, studies demonstrate a reduction in mast cell numbers within the brains of older individuals and a similar decline in dermal mast cells.^66^ In contrast, no significant differences were found between older and younger subjects in mast cell populations in the lamina propria of the jejunum; however, increased mast cell counts were observed in vein samples from older patients. Additionally, investigations involving murine models have revealed variability in mast cell profiles across different tissues and age groups.^67,68^

## Eosinophils

 Eosinophils are pivotal in asthma, particularly in its severe manifestations. Age-related modifications in eosinophil function may considerably affect the severity and clinical expression of asthma in older individuals, underscoring the relevance of targeted therapies against eosinophils for treating severe eosinophilic asthma.

 While research on age-associated changes in eosinophil numbers and functions remains limited, evidence suggests that eosinophils from older adults may exhibit altered effector functions, diminishing their role in airway hyperresponsiveness. Specifically, studies indicate that antigen-sensitized and -challenged aged mice demonstrate greater eosinophilia in the bronchial alveolar lavage fluid (BALF) compared to younger counterparts; however, the degree of airway hyperresponsiveness appears to be reduced in older mice. A study exploring eosinophil function in asthmatic adults aged 55-80 years revealed decreased degranulation in response to interleukin-5 (IL-5) stimulation, accompanied by a trend toward diminished superoxide production in comparison to younger asthmatics aged 20-40 years (6). Conversely, *in-vitro* assessments of eosinophil effector functions, particularly leukotriene C4 production, indicated no significant differences between older and younger asthmatic subjects. Moreover, older asthmatics with indicators of atopy continue to demonstrate sputum eosinophil counts that are comparable to those observed in younger asthmatic individuals.^69^

 The following key points highlight the role of eosinophils in asthma concerning aging:

 - Eosinophilia levels in the airway are similar across various age groups of asthmatics.

 - Significant variations in peripheral blood eosinophil effector functions correlate with age-related changes.^6,9^ Eosinophils also contribute to airway remodeling and the overall inflammatory processes within the airways. Eosinophilic airway inflammation is a hallmark of asthma.^33^ It is noteworthy that neutrophilic asthma is more prevalent than eosinophilic asthma. Adult-onset asthma tends to show a higher prevalence among females, whereas eosinophilic asthma exhibits a distinctive onset distribution, typically occurring between the ages of 25 and 35.

 Furthermore, recent advancements in biologics targeting eosinophils have been developed, providing safe and effective alternatives to oral corticosteroids, thereby creating new avenues for treatment in patients with severe eosinophilic asthma.^70^

## Innate Iymphoid Cell (ILC)

 Since their initial identification in 2010, the field of research on innate lymphoid cells (ILCs), particularly ILC2s, has seen significant advancements. In mouse models, ILC2s have been observed to exhibit both pathological and protective roles during allergic immune responses induced by pathogens or allergens, primarily through the secretion of type 2 cytokines and amphiregulin, respectively.^71^ ILC2s interact synergistically with an array of immune cells, such as CD4 + T cells, macrophages, eosinophils, natural killer T (NKT) cells, and epithelial cells, to promote allergic airway inflammation. However, the role of ILC2s in chronic and episodic airway inflammation in human asthma, as well as in other allergic conditions such as atopic dermatitis and chronic rhinosinusitis with nasal polyps, remains an area of active investigation.

 A comprehensive understanding of the diverse contributions of ILC types and their molecular mechanisms in triggering asthma and other atopic diseases may facilitate the development of novel therapeutic strategies. Ongoing research into the role of ILCs, particularly in the context of aging and asthma, remains crucial. Evidence suggests that ILCs are implicated in asthma pathogenesis and exhibit distinct roles across various asthma phenotypes. Although the specific impact of ILCs on aging relative to asthma is yet to be fully elucidated, it is acknowledged that ILCs, especially ILC2s, may play a critical role in the initiation of asthma, even in the absence of adaptive immune responses. Continuous exploration of the involvement of different ILC subsets and the molecular mechanisms underlying their activation in asthma and other atopic conditions may potentially lead to new therapeutic interventions addressing this significant public health issue.^72^

## Epigenetic Mechanisms and Asthma in Older Adults

 Emerging evidence indicates that epigenetic regulation contributes significantly to asthma pathogenesis in older adults, complementing structural and immunological changes driven by aging. Epigenetic mechanisms (including DNA methylation, histone modifications, and non-coding RNAs) modulate gene expression in response to environmental and inflammatory stimuli, thereby influencing innate and adaptive immunity.

## DNA Methylation and Epigenetic Aging

 Epigenome-wide studies have identified altered methylation profiles in asthmatic patients, many involving genes linked to airway inflammation and immune regulation. Accelerated “epigenetic aging,” measured by DNA methylation clocks, correlates with systemic inflammation and reduced lung function, suggesting that age-related methylation drift exacerbates asthma severity and contributes to corticosteroid resistance in elderly phenotypes.^73^

## Histone Modifications and Trained Immunity

 Histone modifications alter chromatin accessibility, enabling persistent activation of inflammatory pathways. In airway macrophages and monocytes, such changes underpin trained immunity, a process that enhances long-term innate responsiveness. In older adults, this reprogramming may amplify IL-6, TNF-α, and IL-8 production, reinforcing the low-grade inflammation characteristic of “inflamm-aging” and shaping non-allergic asthma phenotypes.^74^

## Non-Coding RNAs

 MicroRNAs and other non-coding RNAs regulate post-transcriptional gene expression and have been linked to airway remodeling and cytokine imbalance in asthma. Age-associated alterations in miRNA expression provide an additional layer of immune dysregulation and hold potential as minimally invasive biomarkers of disease activity.

## Clinical Relevance

 These findings highlight epigenetic changes as both mechanistic drivers and potential biomarkers of asthma in later life. DNA methylation signatures, miRNA panels, and histone marks may inform age-specific risk stratification and monitoring. Moreover, because epigenetic modifications are reversible, therapeutic approaches such as histone deacetylase inhibitors, metabolic modulators, or lifestyle-based interventions merit further exploration.

 In sum, asthma in older adults reflects not only immunosenescence and structural decline but also dynamic epigenetic remodeling. Integrating epigenomic profiling into clinical studies will clarify whether these changes act as causal factors or consequences of disease and may open new avenues for precision management in aging populations.

**Figure 1:**
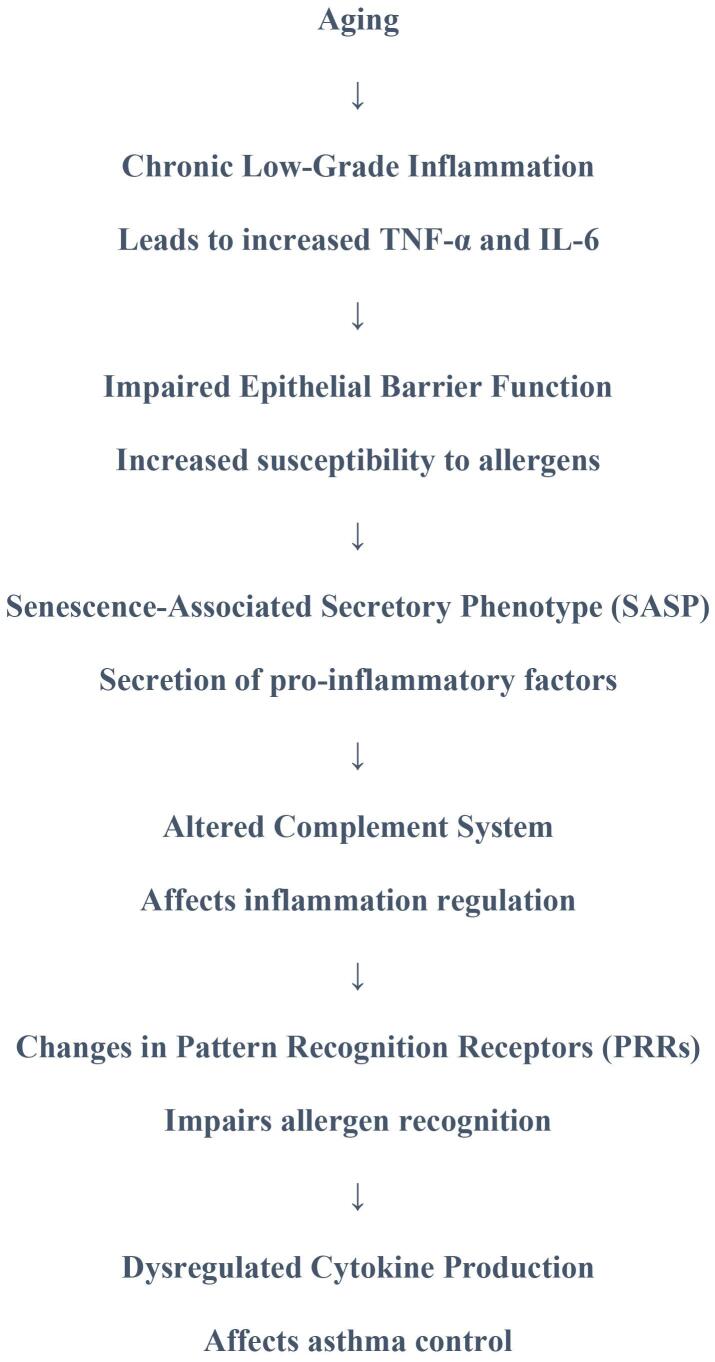
Flowchart of the Impact of Aging on Inflammatory Pathways, Barrier Function, and Asthma Outcomes

 An overview of the interconnected processes discussed is illustrated in the flowchart in (Figure 1).

**Figure 2:**
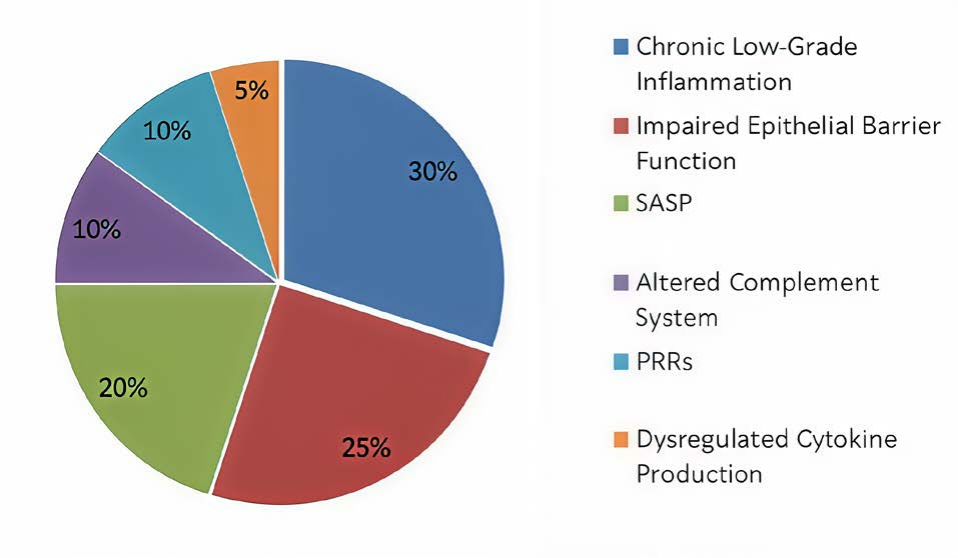
Relative Importance of Aging-Related Factors in Asthma Management for Older Adults

 The key factors related to aging and immune system changes in asthma among older adults, along with relative percentages that reflect their importance are shown in Figure 2.

## Discussion

 The recognition and understanding of late-onset asthma in older adults have garnered increasing attention due to the distinct pathophysiology, clinical phenotypes, comorbidity profiles, and treatment challenges associated with this population.^75^ A multitude of research gaps exist in the study of asthma in older adults, including the fact that asthma in this population is often underdiagnosed and undertreated, highlighting the need for better recognition and management strategies. It is also possible that asthma in older people has different phenotypes compared with younger people, which may affect diagnosis and treatment.^76^

 The presence of underlying diseases, the existence of concomitant age-related health problems Including Perceived Stress Scale (PSS), and the psychosocial effects of aging have the potential to complicate the diagnosis and clinical manifestations of asthma in the aging population. Conversely, the underlying mechanisms of asthma in older individuals may differ from those observed in younger individuals, which could facilitate the development of targeted therapeutic interventions.^77,78^

 A salient feature of elderly asthma patients is the predominance of neutrophilic airway inflammation, a phenomenon that contrasts with the eosinophilic profiles observed in younger cohorts. This shift is accompanied by augmented activity of ILC2s and epithelial cytokines such as IL-33 and thymic stromal lymphopoietin (TSLP), which influence the burst of type 2 cytokines (IL-5, IL-13).^79^

 Comprehensive biomarker profiling studies have identified an array of serum markers in elderly asthmatics, including IL-6, IL-8, IL-17, TNF-α, YKL-40, and serum amyloid A, which index the complex interplay of inflammation, airway remodeling, and immunosenescence. The elderly asthmatic population is characterized by a high burden of comorbidities, including cardiovascular diseases, diabetes mellitus, depression, obesity, gastroesophageal reflux disease (GERD), and cognitive impairment. The significance of this comorbidity burden is multifaceted and has significant implications for healthcare resources and outcomes.^75^

 The diagnosis and treatment of asthma in older adults poses distinctive challenges due to the considerable physiological and immunological alterations that accompany the aging process. Late-onset asthma in older adults is increasingly acknowledged as a distinct clinical entity from early-onset asthma, exhibiting varying immunologic, physiologic, and epidemiological attributes.^33^ Late-onset asthma in the elderly population is distinct from early-onset asthma, often characterized by lower atopy prevalence, reduced eosinophilic inflammation, and more pronounced fixed airflow obstruction. The immune system in older individuals undergoes “immunosenescence,” which includes a decline in adaptive immune responses and maladaptations in the innate immune system. These include diminished mucociliary clearance and skewed cytokine production, leading to a phenomenon referred to as “inflamm-aging”. This chronic low-grade inflammatory state, typified by elevated pro-inflammatory cytokines such as IL-6, TNF-α, and IL-8, frequently culminates in neutrophilic airway inflammation, contrasting with the eosinophilic profiles typically observed in younger patients.^80,81^

 Biomarker profiling has become increasingly vital, as elevated serum levels of biomarkers such as eotaxin-2, TGF-β1, interleukin-6, interleukin-8, interleukin-17, TNF-α, YKL-40, and serum amyloid A indicate inflammation, airway remodeling, and immunosenescence.^82^ These biomarkers can facilitate the delineation of specific phenotypic subgroups within the elderly asthma population, thereby transcending the conventional Th2/non-Th2 paradigm and paving the way for personalized medicine. The presence of significant comorbidities, including cardiovascular diseases, diabetes, depression, obesity, and gastroesophageal reflux disease (GERD), further complicates diagnosis and management, necessitating a comprehensive assessment that considers the patient’s overall health status.^79,83^

 Treatment strategies for late-onset asthma in the elderly must account for these complex factors, including the predisposition to corticosteroid resistance and an increased frequency of exacerbations, often triggered by viral infections and environmental pollutants. The severity of late-onset asthma frequently necessitates higher doses of inhaled corticosteroids or alternative therapeutic approaches, such as biologics, especially in cases with persistent inflammation or specific biomarker profiles. The management of asthma in older adults necessitates a comprehensive approach that addresses the high prevalence of comorbidities. These conditions have the potential to exert a substantial influence on the effectiveness of treatment regimens and the ensuing outcomes for patients. Development of a comprehensive and individualized treatment plan is imperative to enhance the quality of life and alleviate the healthcare burden experienced by elderly individuals afflicted with asthma.^84^

## Conclusion

 Although new data in asthma and immunology emerge continuously, synthesizing this evidence remains essential to address unresolved questions and guide future researc. Asthma in older adults is shaped by immunosenescence, chronic low-grade inflammation, epithelial dysfunction, and altered innate immune signaling, resulting in clinical phenotypes that differ substantially from those of younger patients. Biomarkers reflecting neutrophilic inflammation, cytokine dysregulation, innate immune receptor activity, and epigenetic aging are increasingly supported by research from established academic centers as tools for refining disease classification and management.

 Future studies should prioritize longitudinal, age-stratified, and multi-omics approaches to validate these biomarkers and clarify causal mechanisms. Integrating innate immune profiling with clinical phenotypes will be essential for advancing precision medicine strategies in elderly asthma. In summary, while the field continues to evolve rapidly, this review provides a concise and evidence-based synthesis that may assist clinicians and researchers in navigating current knowledge and identifying priorities for future investigation.

## References

[1] Rahimian N, Aghajanpour M, Jouybari L, Ataee P, Fathollahpour A, Lamuch-Deli N (2021). The prevalence of asthma among Iranian children and adolescent: a systematic review and meta-analysis. Oxid Med Cell Longev.

[2] Busse PJ, Birmingham JM, Calatroni A, Manzi J, Goryachokovsky A, Fontela G, et al. Effect of aging on sputum inflammation and asthma control. J Allergy Clin Immunol 2017;139(6):1808-18.e6. doi: 10.1016/j.jaci.2016.09.015. PMC5385166 27725186

[3] Battaglia S, Benfante A, Spatafora M, Scichilone N (2016). Asthma in the elderly: a different disease?. Breathe (Sheff).

[4] Meiners S, Eickelberg O, Königshoff M (2015). Hallmarks of the ageing lung. Eur Respir J.

[5] Gao H, Nepovimova E, Adam V, Heger Z, Valko M, Wu Q (2024). Age-associated changes in innate and adaptive immunity: role of the gut microbiota. Front Immunol.

[6] Busse PJ, Mathur SK (2010). Age-related changes in immune function: effect on airway inflammation. J Allergy Clin Immunol.

[7] Wang J, Zhang X, Zhang L, Liu Y, Wang G, Zhang HP, et al. Age-related clinical characteristics, inflammatory features, phenotypes, and treatment response in asthma. J Allergy Clin Immunol Pract 2023;11(1):210-9.e3. doi: 10.1016/j.jaip.2022.09.029. 36191867

[8] Murray MA, Chotirmall SH (2015). The impact of immunosenescence on pulmonary disease. Mediators Inflamm.

[9] Busse PJ, Mathur SK (2010). Age-related changes in immune function: effect on airway inflammation. J Allergy Clin Immunol.

[10] Wenzel SE (2012). Asthma phenotypes: the evolution from clinical to molecular approaches. Nat Med.

[11] de Andrade Belitardo EMM, Almeida PCA, Sena F, da Silva ES, Rocha DJ, dos Santos Nano JM (2025). A treatment-resistant severe asthma phenotype with dysregulated Hippo pathway as shown by sputum transcriptomics and proteomics. Allergies.

[12] Bailey KL (2022). Aging diminishes mucociliary clearance of the lung. Adv Geriatr Med Res.

[13] Agusti A, Faner R (2019). Lung function trajectories in health and disease. Lancet Respir Med.

[14] Schleich F, Graff S, Guissard F, Henket M, Paulus V, Louis R (2021). Asthma in elderly is characterized by increased sputum neutrophils, lower airway caliber variability and air trapping. Respir Res.

[15] Simpson JL, Grissell TV, Douwes J, Scott RJ, Boyle MJ, Gibson PG (2007). Innate immune activation in neutrophilic asthma and bronchiectasis. Thorax.

[16] Soma T, Nagata M (2022). Immunosenescence, inflammaging, and lung senescence in asthma in the elderly. Biomolecules.

[17] Shaw DE, Sousa AR, Fowler SJ, Fleming LJ, Roberts G, Corfield J (2015). Clinical and inflammatory characteristics of the European U-BIOPRED adult severe asthma cohort. Eur Respir J.

[18] Christenson S. The microbiome in asthma, COPD, and asthma-COPD overlap. In: Asthma, COPD, and Overlap. Boca Raton, FL: CRC Press; 2018. p. 95-106.

[19] Clemente-Suárez VJ, Mielgo-Ayuso J, Ramos-Campo DJ, Beltran-Velasco AI, Martínez-Guardado I, Navarro Jimenez E (2023). Basis of preventive and non-pharmacological interventions in asthma. Front Public Health.

[20] Walters MS, De BP, Salit J, Buro-Auriemma LJ, Wilson T, Rogalski AM (2014). Smoking accelerates aging of the small airway epithelium. Respir Res.

[21] Kermani NZ, Adcock IM, Djukanović R, Chung F, Schofield JPR (2023). Systems biology in asthma. Adv Exp Med Biol.

[22] Gautam Y, Johansson E, Mersha TB (2022). Multi-omics profiling approach to asthma: an evolving paradigm. J Pers Med.

[23] Sigari N, Mohammadi S, Afkhamzadeh AR (2010). A comparison of serum CRP levels measured by a highly sensitive method in healthy subjects and asthmatic patients. Sci J Kurdistan Univ Med Sci.

[24] Olsthoorn SEM, van Krimpen A, Hendriks RW, Stadhouders R (2025). Chronic inflammation in asthma: looking beyond the Th2 cell. Immunol Rev.

[25] Tizazu AM, Mengist HM, Demeke G (2022). Aging, inflammaging and immunosenescence as risk factors of severe COVID-19. Immun Ageing.

[26] Pelletier M, Maggi L, Micheletti A, Lazzeri E, Tamassia N, Costantini C (2010). Evidence for a cross-talk between human neutrophils and Th17 cells. Blood.

[27] Khosa JK, Louie S, Lobo Moreno P, Abramov D, Rogstad DK, Alismail A (2023). Asthma care in the elderly: practical guidance and challenges for clinical management - a framework of 5 “Ps”. J Asthma Allergy.

[28] Columbo M, Wong B, Panettieri RA Jr, Rohr AS (2013). Asthma in the elderly: the role of exhaled nitric oxide measurements. Respir Med.

[29] Miller AL (2001). The etiologies, pathophysiology, and alternative/complementary treatment of asthma. Altern Med Rev.

[30] Wang Z, Pianosi P, Keogh K, Zaiem F, Alsawas M, Alahdab F, et al. AHRQ Comparative Effectiveness Reviews. The Clinical Utility of Fractional Exhaled Nitric Oxide (FeNO) in Asthma Management. Rockville, MD: Agency for Healthcare Research and Quality (US); 2017. 29533572

[31] Zhang Y. Encyclopedia of Global Health. London: SAGE Publications; 2008.

[32] Skloot GS, Busse PJ, Braman SS, Kovacs EJ, Dixon AE, Vaz Fragoso CA (2016). An official American Thoracic Society workshop report: evaluation and management of asthma in the elderly. Ann Am Thorac Soc.

[33] de Vries M, Nwozor KO, Muizer K, Wisman M, Timens W, van den Berge M (2022). The relation between age and airway epithelial barrier function. Respir Res.

[34] Aghapour M, Raee P, Moghaddam SJ, Hiemstra PS, Heijink IH (2018). Airway epithelial barrier dysfunction in chronic obstructive pulmonary disease: role of cigarette smoke exposure. Am J Respir Cell Mol Biol.

[35] Sigari N, Jalili A, Mahdawi L, Ghaderi E, Shilan M (2016). Soluble CD93 as a novel biomarker in asthma exacerbation. Allergy Asthma Immunol Res.

[36] Angelidis I, Simon LM, Fernandez IE, Strunz M, Mayr CH, Greiffo FR (2019). An atlas of the aging lung mapped by single cell transcriptomics and deep tissue proteomics. Nat Commun.

[37] Rojas M, Meiners S, Le Saux CJ. Molecular Aspects of Aging: Understanding Lung Aging. Hoboken: John Wiley & Sons; 2014.

[38] Lambrecht BN, Hammad H (2015). The immunology of asthma. Nat Immunol.

[39] Holgate ST (2012). Innate and adaptive immune responses in asthma. Nat Med.

[40] Chelvarajan RL, Liu Y, Popa D, Getchell ML, Getchell TV, Stromberg AJ (2006). Molecular basis of age-associated cytokine dysregulation in LPS-stimulated macrophages. J Leukoc Biol.

[41] De Maeyer RPH, Sikora J, Bracken OV, Shih B, Lloyd AF, Peckham H, et al. Age-associated inflammatory monocytes are increased in menopausal females and reversed by hormone replacement therapy. bioRxiv [Preprint]. April 1, 2025. Available from: https://www.biorxiv.org/content/10.1101/2025.03.28.645904v1. 10.1111/acel.70249 PMC12611317 41064932

[42] Liu T, Zhou YT, Wang LQ, Li LY, Bao Q, Tian S, et al. NOD-like receptor family, pyrin domain containing 3 (NLRP3) contributes to inflammation, pyroptosis, and mucin production in human airway epithelium on rhinovirus infection. J Allergy Clin Immunol 2019;144(3):777-87.e9. doi: 10.1016/j.jaci.2019.05.006. 31102698

[43] Wang MM, Lu M, Zhang CL, Wu X, Chen JX, Lv WW (2018). Oxidative stress modulates the expression of toll-like receptor 3 during respiratory syncytial virus infection in human lung epithelial A549 cells. Mol Med Rep.

[44] Wu Y, Goplen NP, Sun J (2021). Aging and respiratory viral infection: from acute morbidity to chronic sequelae. Cell Biosci.

[45] Panda A, Qian F, Mohanty S, van Duin D, Newman FK, Zhang L (2010). Age-associated decrease in TLR function in primary human dendritic cells predicts influenza vaccine response. J Immunol.

[46] Huang HJ, Lin YL, Liu CF, Kao HF, Wang JY (2011). Mite allergen decreases DC-SIGN expression and modulates human dendritic cell differentiation and function in allergic asthma. Mucosal Immunol.

[47] Feng Y, Liu X, Wang Y, Du R, Mao H (2023). Delineating asthma according to inflammation phenotypes with a focus on paucigranulocytic asthma. Chin Med J (Engl).

[48] Schleich FN, Manise M, Sele J, Henket M, Seidel L, Louis R (2013). Distribution of sputum cellular phenotype in a large asthma cohort: predicting factors for eosinophilic vs neutrophilic inflammation. BMC Pulm Med.

[49] Hough KP, Curtiss ML, Blain TJ, Liu RM, Trevor J, Deshane JS (2020). Airway remodeling in asthma. Front Med (Lausanne).

[50] Chilvers ER, Cadwallader KA, Reed BJ, White JF, Condliffe AM (2000). The function and fate of neutrophils at the inflamed site: prospects for therapeutic intervention. J R Coll Physicians Lond.

[51] Fortin CF, Lesur O, Fulop T Jr (2007). Effects of aging on triggering receptor expressed on myeloid cells (TREM)-1-induced PMN functions. FEBS Lett.

[52] Larbi A, Franceschi C, Mazzatti D, Solana R, Wikby A, Pawelec G (2008). Aging of the immune system as a prognostic factor for human longevity. Physiology (Bethesda).

[53] Gomez CR, Nomellini V, Faunce DE, Kovacs EJ (2008). Innate immunity and aging. Exp Gerontol.

[54] Allen NC, Reyes NS, Lee JY, Peng T (2022). Intersection of inflammation and senescence in the aging lung stem cell niche. Front Cell Dev Biol.

[55] Lehrer RI, Ganz T, Selsted ME, Babior BM, Curnutte JT (1988). Neutrophils and host defense. Ann Intern Med.

[56] Moss CE, Phipps H, Wilson HL, Kiss-Toth E (2023). Markers of the ageing macrophage: a systematic review and meta-analysis. Front Immunol.

[57] van der Veen TA, de Groot LES, Melgert BN (2020). The different faces of the macrophage in asthma. Curr Opin Pulm Med.

[58] Bachstetter AD, Van Eldik LJ (2010). The p38 MAP kinase family as regulators of proinflammatory cytokine production in degenerative diseases of the CNS. Aging Dis.

[59] Fricker M, Gibson PG (2017). Macrophage dysfunction in the pathogenesis and treatment of asthma. Eur Respir J.

[60] Matangkasombut P, Pichavant M, Dekruyff RH, Umetsu DT (2009). Natural killer T cells and the regulation of asthma. Mucosal Immunol.

[61] Zhang Y, Wallace DL, de Lara CM, Ghattas H, Asquith B, Worth A (2007). In vivo kinetics of human natural killer cells: the effects of ageing and acute and chronic viral infection. Immunology.

[62] Joyce S (2001). CD1d and natural T cells: how their properties jump-start the immune system. Cell Mol Life Sci.

[63] Lezmi G, Leite-de-Moraes M (2018). Invariant natural killer T and mucosal-associated invariant T cells in asthmatic patients. Front Immunol.

[64] Mocchegiani E, Muzzioli M, Giacconi R, Cipriano C, Gasparini N, Franceschi C (2003). Metallothioneins/PARP-1/IL-6 interplay on natural killer cell activity in elderly: parallelism with nonagenarians and old infected humans Effect of zinc supply. Mech Ageing Dev.

[65] Méndez-Enríquez E, Hallgren J (2019). Mast cells and their progenitors in allergic asthma. Front Immunol.

[66] Arranz E, O’Mahony S, Barton JR, Ferguson A (1992). Immunosenescence and mucosal immunity: significant effects of old age on secretory IgA concentrations and intraepithelial lymphocyte counts. Gut.

[67] Pascual G, Mendieta C, García-Honduvilla N, Corrales C, Bellón JM, Buján J (2007). TGF-beta1 upregulation in the aging varicose vein. J Vasc Res.

[68] Atsuta R, Akiyama K, Shirasawa T, Okumura K, Fukuchi Y, Ra C (1999). Atopic asthma is dominant in elderly onset asthmatics: possibility for an alteration of mast cell function by aging through Fc receptor expression. Int Arch Allergy Immunol.

[69] Shen K, Zhang M, Zhao R, Li Y, Li C, Hou X (2023). Eosinophil extracellular traps in asthma: implications for pathogenesis and therapy. Respir Res.

[70] Kawamatawong T, Siripongpun S, Rerkpattanapipat T (2016). Role of eosinophilic inflammation and atopy in elderly asthmatic patients. Asia Pac Allergy.

[71] Wallrapp A, Riesenfeld SJ, Burkett PR, Kuchroo VK (2018). Type 2 innate lymphoid cells in the induction and resolution of tissue inflammation. Immunol Rev.

[72] Kim HY, Umetsu DT, Dekruyff RH (2016). Innate lymphoid cells in asthma: will they take your breath away?. Eur J Immunol.

[73] Vasileva D, Greenwood CMT, Daley D (2023). A review of the epigenetic clock: emerging biomarkers for asthma and allergic disease. Genes (Basel).

[74] Sheikhpour M, Maleki M, Ebrahimi Vargoorani M, Amiri V (2021). A review of epigenetic changes in asthma: methylation and acetylation. Clin Epigenetics.

[75] Nikolova D, Todorova Y, Hammoudeh Z, Rukova B, Emilova R, Aleksova M (2025). Gene expression changes as biomarkers of immunosenescence in Bulgarian individuals of active age. Biomedicines.

[76] Yáñez A, Cho SH, Soriano JB, Rosenwasser LJ, Rodrigo GJ, Rabe KF (2014). Asthma in the elderly: what we know and what we have yet to know. World Allergy Organ J.

[77] Hanania NA, King MJ, Braman SS, Saltoun C, Wise RA, Enright P (2011). Asthma in the elderly: current understanding and future research needs--a report of a National Institute on Aging (NIA) workshop. J Allergy Clin Immunol.

[78] Maroufizadeh S, Zareiyan A, Sigari N (2014). Reliability and validity of Persian version of perceived stress scale (PSS-10) in adults with asthma. Arch Iran Med.

[79] Hirano T, Matsunaga K (2018). Late-onset asthma: current perspectives. J Asthma Allergy.

[80] Fulop T, Larbi A, Pawelec G, Khalil A, Cohen AA, Hirokawa K (2023). Immunology of aging: the birth of inflammaging. Clin Rev Allergy Immunol.

[81] Dunn RM, Busse PJ, Wechsler ME (2018). Asthma in the elderly and late-onset adult asthma. Allergy.

[82] Zhang T, Huang Y, Ji X, Wu T, Xiao P (2025). CCL11 (Eotaxin) promotes the advancement of aging-related cardiovascular diseases. Rev Cardiovasc Med.

[83] Lima-Silva ML, Torres KCL, de Melo Mambrini JV, Brot NC, Santos SO, Martins-Filho OA (2024). A nationwide study on immunosenescence biomarkers profile in older adults: ELSI-Brazil. Exp Gerontol.

[84] Ji H, Tan LD, Hafzalla GW, Nguyen N, Alismail A (2024). Navigating biologic therapies in elderly asthma. Respir Med.

